# Barriers and facilitators to implementing a process to enable parent escalation of care for the deteriorating child in hospital

**DOI:** 10.1111/hex.12806

**Published:** 2018-07-02

**Authors:** Fenella J. Gill, Gavin D. Leslie, Andrea P. Marshall

**Affiliations:** ^1^ School of Nursing, Midwifery and Paramedicine Faculty of Health Sciences Curtin University Perth WA Australia; ^2^ Perth Children's Hospital Child and Adolescent Health Services Perth WA Australia; ^3^ School of Nursing and Midwifery Clinical Chair Gold Coast Health Southport Qld Australia; ^4^ Centre for Health Practice Innovation Menzies Health Institute Queensland Southport Qld Australia; ^5^ School of Nursing and Midwifery Griffith University Southport Qld Australia; ^6^ Gold Coast Hospital and Health Service Gold Coast University Hospital Southport Qld Australia; ^7^ Nursing and Midwifery Education and Research Unit Southport Qld Australia

**Keywords:** clinical deterioration, family escalation of care, implementation, paediatric, parent

## Abstract

**Objective:**

To identify barriers and facilitators to implementing a parent escalation of care process: Calling for Help (C4H).

**Design:**

Audits, semi‐structured interviews and focus groups guided by the Theoretical Domains Framework.

**Setting:**

Australian paediatric hospital where a parent escalation of care process was introduced in the previous 6 months.

**Participants:**

Four children, 13 parents, 91 nurses and doctors including Medical Emergency Team (MET) members.

**Main outcome measures:**

Parent awareness and involvement in escalating care and factors impacting implementation of C4H.

**Results:**

Two audits identified low level of parent awareness (16/88, 19% and 5/85, 6%). Parent involvement in escalation of care was documented in 11/62 (18%) events. The main facilitators included uniformly positive views that C4H was in the child's best interest, acknowledgement that parents had skills to recognize deterioration and would take action. C4H was considered to add to patient safety and being reviewed by the MET was a patient benefit. Key barriers were the low level of awareness, doubt about parent capabilities, concern about parents’ information overload, anticipated overuse of resources, staff unease about possible repercussions and anticipated difficulty for parents to question staff with potential negative effects on parent‐staff relationships. Overall C4H presents a challenge to traditional hospital hierarchy and culture.

**Conclusions:**

Although there was a low level of awareness about C4H in practice, there was in‐principle support for the concept. Initial strategies had primarily targeted policy change without taking into account the need for practice and organizational behaviour changes. Using a theoretical approach to identify key factors will enable a targeted approach to implementation.

## 1 INTRODUCTION

The concept of family‐centred care is based on the belief that in health care, a child's developmental and socio‐emotional needs are best met when the family are involved in planning, delivering and evaluating care.^1^ The important role that families contribute to patient outcomes extends to the family's contribution in the early detection of patient clinical deterioration. Rapid Response Systems aim to improve the detection and management of deterioration for patients in hospital.^2^ Although families do not have responsibility for the formal assessment of clinical changes in patient condition, their familiarity and continued presence with their child place them at an advantage in recognizing early signs of deterioration.

How family involvement in Rapid Response Systems has been implemented and evaluated in the USA and UK^3^, ^4^, ^5^ context was the focus of three recent reviews. Evaluations reported uniformly positive family satisfaction.^6^, ^7^, ^8^, ^9^, ^10^ Families were happy that there was a process in place for them to voice concerns, irrespective of whether they initiated an escalation of care call or not. Health‐care professional attitudes have been reported as barriers to family‐initiated escalation of care. Concerns have been raised that family‐initiated escalation of care calls would result in numerous calls for non‐urgent situations^11^; about possible repercussions following family escalation care for a patient they were responsible for^9^; feeling loss of control; becoming deskilled; and health‐care professionals’ decision making being undermined by families.^10^ Interestingly, following introduction of family escalation of care programmes, an increased number of staff escalation calls have been noted,^8^, ^12^, ^13^ possibly indicating behaviour change by staff when they responded to family concerns. None of the articles described any guidelines for intervention development, implementation and evaluation or used a theoretical foundation. There is no research reporting the most effective way to involve families in escalation of care.^14^

A process for parent escalation of care was introduced at the paediatric hospital in Western Australia in 2015 and it was opportune to evaluate the effectiveness of implementation and sustainability. This study is part of the PARTNER Project which examined and addressed implementation issues organized in three phases: Phase 1—identification of barriers and facilitators to implementation, Phase 2—implementing a revised parent escalation of care process and Phase 3—evaluation. The Theoretical Domains Framework^15^ and an Integrated Knowledge Translation^16^ approach involved establishing the Study Steering Group (consisting of researchers, clinicians and consumers) to inform the approach to and guide the conduct of the study. This paper reports on Phase 1: examining the implementation of the process for parent escalation of care to identify barriers and facilitators.

## 2 METHODS

### 2.1 Setting

The study site is a specialist paediatric facility offering a full range of services to children from 0 to 16 years. A two‐tiered Rapid Response System consisted of a Medical Emergency Team (MET) call when early recognition and response to clinical deterioration were triggered and a Code Blue call when a resuscitation response was required. The Children's Early Warning Tool^17^ is an age‐specific observation chart for monitoring vital signs and oxygen therapy which are calculated into a composite score between 1 and 20 which is then used as a trigger to escalate care if the score is increasing. For example, a score between 4 and 5 requires notification of the nurse in charge and a medical review, a score between 6 and 7 requires a more senior medical review within 15 minutes and notification of the treating physician, and a score of 8 or greater necessitates an emergency call with a MET response within 5 mins.

**Figure 1:**
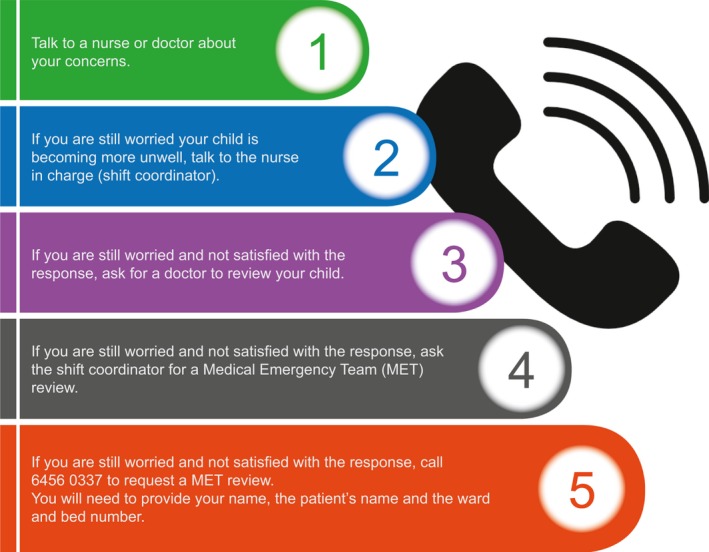
Calling for Help 5 steps

In May 2015, the locally developed parent escalation of care process, named Calling for Help (C4H), was introduced, consisting of updates to the existing Rapid Response System policies and guidelines and distribution of parent information brochures. The C4H process was developed in consultation with the hospital Consumer Advisory Council and involved five steps for parents to incrementally escalate their concerns (see Figure 1).

### 2.2 Design

Any implementation research aiming to generate knowledge that is generalizable should be guided by theory, yet few theory‐based implementation studies have been reported.^15^ This study included both knowledge translation and implementation science methodologies. The SQUIRE 2.0 guidelines^18^ for reporting new knowledge about how to improve health care were followed. The study was driven by the Theoretical Domains Framework,^15^ developed using an expert consensus and validation process to identify and synthesize psychological and organizational theory influencing health practitioner behaviour. The Theoretical Domains Framework is organized into 14 domains covering factors that influence health professionals’ behaviour and behaviour change. This framework provides an extensive framework to theoretically investigate implementation issues and systematically inform practice change interventions.^19^, ^20^, ^21^

### 2.3 Data collection

**Figure 2:**
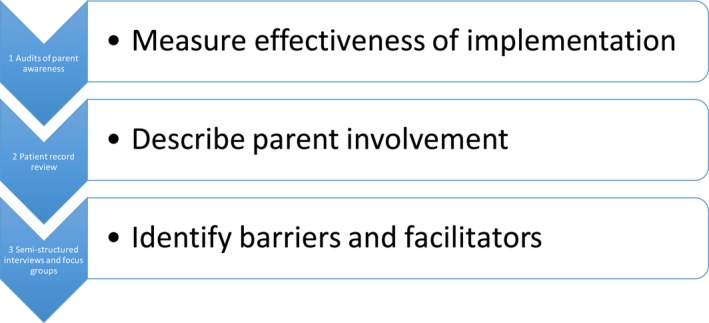
Data collection methods

Parent escalation of care was measured using the level of parent awareness about the process and the rate of activation.^22^ Following introduction of the C4H process in May 2015, data were collected over 8 months using three sources to (i) measure parent awareness of C4H to understand the effectiveness of implementation, (ii) describe how parents were involved in escalation of care, and (iii) identify barriers and facilitators for parent involvement in escalation of care (see Figure 2). Facility Human Research Ethics Committees approved the study protocol.

#### 2.3.1 Parent audit

Two audits were conducted (4 weeks apart) to identify the level of parent awareness about the C4H process. These were performed by asking all parents who accompanied their child in inpatient areas on the days of the audits if they were aware of the C4H. Parents were also asked how they would escalate their concerns if worried about their child's medical condition.

#### 2.3.2 Patient record review

Health records of patients who had received MET calls in the 6 months following C4H introduction were analysed to describe their characteristics and identify how parents were involved in escalation of care in the eight hours prior to the MET call being placed.

#### 2.3.3 Interviews

Stakeholder interviews and focus groups were conducted using a semi‐structured interview guide developed using the Theoretical Domains Framework, tested and refined prior to data collection (Supporting Information—called PARTNER project interview guide). The lead researcher (a female PhD‐prepared nurse researcher experienced in conducting interviews and focus groups) conducted all staff interviews and focus groups and supervised the parent and child interviews. She was known to some staff participants as a staff member but did not work with or have influence on their work. She had no prior relationship with any of the parent or child participants. The duration of the interviews and focus groups was between 20 and 60 minutes.

Participants were (i) parents of patients who had received a MET call during their stay in hospital were contacted by mail and telephoned with an invitation to participate, (ii) children aged at least 12 years who had experienced an inpatient admission for greater than 48 hours were recruited by advertising in patient areas and through clinical nurse managers of inpatient areas identifying potential participants, and (iii) nurses and doctors were recruited by advertising through the hospital email, newsletters and scheduling focus groups for ward nurses.

### 2.4 Data analysis

Patient and participant characteristics and audit results were collated using descriptive statistics. The COREQ checklist for interviews and focus groups^23^ and standards for reporting qualitative research^24^ guided the qualitative study reporting. Interviews and focus groups were audio‐recorded and transcribed verbatim. Qualitative data analysis was undertaken using the framework approach.^25^, ^26^, ^27^ This methodological approach enabled the systematic integration of data driven codes with theory‐driven categories and allowed the Theoretical Domains Framework to be integral to the process (Table 1). Collection and analysis of data were iterative processes which allowed emerging themes to be further explored in subsequent interviews and focus groups. A second member of the research team checked and confirmed coding of data. A third team member resolved any coding disagreements. The researchers’ interpretation of the findings from the multiple data sources was presented and confirmed with the Study Steering Group.

**Table 1:** Framework approach and data analysis steps

Step 1 Preliminary analysis: The initial immersion in the raw data to become familiar with the diversity and range of the data while noting emerging and recurrent themes.	Reading the transcripts of individual or focus group interviews. Making note of themes emerging from the data on initial reading in relation to the aims of the study.
Step 2 Establishing the thematic framework: The identification of a framework well suited to the examination and referencing of the data.	The “a priori” identification of the TDF as an appropriate thematic framework with which to explore key behavioural constructs and context related to behaviour change. Exploration of the data confirming the appropriateness of the framework to capture recurring themes and issues arising from the respondent's interviews.
Step 3 Indexing: The systematic application of the framework to the textual data.	Identification of sections of relevant text within the transcripts, applying identifying codes and a descriptive label. Preliminary sorting of text within a dedicated framework for each participant group.
Step 4 Charting: The creation of individual charts which capture data related to each of component of the framework.	Combined nurses, doctors, MET responder, parent and children's data sorted into individual charts corresponding to 8 appropriate domains.
Step 5 Mapping and interpretation: The final stage of analysis undertaken to describe the phenomena in detail and review the salience of issues and dynamics operating within the practice context.	Identification of major themes and issues related to local practice. The opportunity to compare points of view across professions, professional and social roles undertaken within the hospital. 24 themes emerged across 8 TDF domains: knowledge; skills; social/professional role and identity; beliefs about capabilities; beliefs about consequences; memory, attention and decision processes; environmental context and resources; behavioural regulation.

## 3 RESULTS

### 3.1 Parent audit

Two audits (January 2016 and February 2016) were undertaken over 2 days each. In Audit 1, 16 of 86 (19%) and in Audit 2, 5 of 85 (6%) parents were aware of C4H. This represented a mean value of 12%. Parents who were not aware of the C4H process reported a number of ways they would respond if they were concerned about their child's medical condition. These included press the emergency nurse call button, go to the nurses’ station, speak to the nurse coordinator to request a nurse or doctor review their child and speak with a social worker or directly call the treating medical team.

### 3.2 Patient health record review

There were 62 MET calls for 45 patients; 67% were for complex patients with comorbidities (categorized into pre‐term and associated conditions, congenital cardiac disease, haematological illness or cancer, congenital syndrome, developmental delay, or respiratory disease). Patient mean age was 47 months (SD 49, range 1‐170). The most common admission principle diagnosis was respiratory illness (n = 26), reflecting typical hospital presentation patterns during the winter season. Ten patients received two MET calls, and four patients received three or more MET calls. The most commonly recorded trigger for a MET call was a Children's Early Warning Tool Score of 8 or more (n = 28, 43%). Parent concerns during the 8 hours preceding the MET calls were recorded for 11 (17%) events when patients subsequently had a MET call, for 5 (8%) patients parent concern was a trigger for the MET call. There were no MET calls directly activated by parents.

### 3.3 Interviews

Four children (age 12‐15 years, one male, three females) with illness‐related experiences as inpatients in specialty settings of neurology, diabetes and oncology were interviewed face to face. Of the thirteen parent interviews (all female participants), twelve were conducted by telephone and one was a face‐to‐face interview. An interpreter enabled a telephone interview with one parent. A total of 92 staff, 73 ward (a mix of surgical and medical) nurses (a mix of junior and experienced, almost all Registered Nurses), 10 ward doctors (both junior and specialist level) and 9 MET responders (experienced ICU nurses and paediatric intensive care specialist doctors), agreed to participate and were interviewed either individually or in 1 of 12 focus groups held at the hospital. A second researcher was present for all interviews and focus groups and took field notes. Data saturation was achieved after nine focus groups, after which no new information or themes emerged. Themes were confirmed during the final three ward focus groups.

Twenty‐four themes across nine Theoretical Domains Framework domains were developed. Table 2 presents the Theoretical Domains Framework domains, themes and supporting participant quotes.

**Table 2:** The TDF domains, themes and supporting participant quotes

TDF Domain	Themes	Exemplary participant quotes
Knowledge	Low level of awareness	*I should've done it and … someone told me about it after when I was in ICU … I would do it now, whereas I probably would have never thought to do it before* (P3)
	Uncertainty that C4H had commenced	*We were given about a dozen (brochures) and we were told that no more would be ordered* (WNF1)
	Positive views about C4H	*I think it's a really good idea and like really helpful* (C1) *It then enables a different team… ICU team… to review them and obviously make sure they're getting the right treatment. I think that's obviously the biggest benefit* (P1)
Skills	Parents could recognize what is not normal	*Like when your [blood glucose is] high you're rude and inconsiderate and you don't know what you're saying and you can't really control yourself … a doctor or nurse could just think that's you normally and your parents know that you're not that type of person* (C2)
	Parents could recognize deterioration	*Parents are used to seeing their child well and healthy. We're only used to seeing their children sick so we get desensitised to what's normal and what's not normal* (WNF2)
	Nurses did not want to use a prepared script	*A script… would make it sound too like something's going to happen* (WNF3)
Belief about consequences	C4H added to patient safety	*At first it's kind of just like mum just listen to the nurses, like they're here, they know what they're doing, they're experienced in this stuff. But afterwards you're like, thank God they did listen in the end* (C1) *Anything you can do to close the loop …we're not perfect* (WDI1)
	Potential for inappropriate calls and over use of resources	*It would be like just using it… because you don't like the doctors or you don't like the nurses* (C1) *Parents…that… think that their kids aren't getting the right treatment and… you… don't have the resources for* (P1) *You can predict those people that will probably call it and it won't be because the child's really deteriorated, it might be… that they're not happy with the pain management…* (WNF5)
	Potential repercussions if staff missed deterioration	*I think is going to be a big concern if that is the trend that we've not listened or we've not escalated care ourselves* (WNF5) *I'd feel pretty useless, disappointed* (WNF8)
Belief about capabilities	Parents would be able to escalate care	*If I could call for help the night my daughter went blue I would have called* (P5) *There are parents who know their child well enough that they can see them deteriorating before the obs change or the staff who are new and don't recognise the patient* (MNF2)
	Doubts about parents’ capabilities to recognize and respond to clinical deterioration	*So you don't just get hysterical parents ringing up… when the nurse hasn't given the medication you know 10 minutes later… I think people get very stressed in hospital and we saw some very poor behaviour on the part of parents when we were in hospital* (P1)
Social/professional role and identity	Parents would escalate care even if it upset staff	*If it came down to the wire and I was that worried about him I probably wouldn't really care* (P6)
	Doctors view that informing parents about C4H was nurses’ role	*Who tells them where their bed are, where their kitchen is? Nurses do that…I would have thought it's appropriate to orientate patients with all of that stuff at the same time, with the same person* (WDF)
	Negative effect on parent‐staff relationship	*I reckon it would make them (nurses) feel a bit insecure … a bit like no one really cares anymore about what their opinion is* (C2)
	C4H will change to the health‐care delivery system	*The end result of distrust in the team, in the ward, in the nurses and that would be my fear …afterwards what sort of relationship have you got with the mum and the patient, they no longer trust you. …if it's a last resort really we're not doing our job and …being so out of touch with… the family and child that we profess to be experts in care of…how have we got it to that point?* (WDI1) *Sometimes even if you're trying to say the concern that you've got that doesn't get listened to, so sometimes if they (parents) did it it's actually powerful* (WNF6)
	C4H is a good fit with family‐centred care practices	*If they followed the process properly we would be involved with it…and we would have got them reviewed anyway* (WNF3)
Memory, attention and decision processes	C4H an additional burden for parents at time of admission	*You don't want to overwhelm people … because they can't take on board everything that they get told* (MNF2)
	Multiple communication strategies required including before admission to hospital	Maybe a link … most people have got their phones, apps that sort of stuff (Parent 6) It definitely needs to be verbal because I don't think people would be confident without having that verbalised (P7)
Motivation and goals	C4H is in the child's best interest	*I wouldn't be afraid to ask for help, for my child if I felt they needed to see someone* (P8)
Environmental context and resources	Benefit of being reviewed by the MET	*If your child was that unwell and no one seemed to listen to you, then obviously getting the ICU team is comforting* (P8) … *even if nothing came out if just having the review meant we were on their radar* (P7)
	It may be too difficult for parents to speak up in hospital	*I'm not a very outspoken person… I don't like to cause … ruffled feathers so I probably want to speak to maybe his doctor first or the nurse and see what they thought* (P11)
	C4H is complex to communicate to parents who do not understand English	*They're the ones that I worry about… have they been afforded the same information and are as aware … as English speaking people?* (WNF5)
	C4H not supported by hospital leaders	*There needs to be someone …to tell us that from this day you're going to start teaching parents on admission …otherwise it's just going to be inconsistent* (WNF3)
Behavioural regulation	Difficult to bypass the traditional hospital culture	*It would take a pretty…, tough assertive mum, dad to get all the way down to step 5 and call* (WNF2)

C, Child; MET, Medical Emergency Team; MNF, Nurse Focus Group; P, Parent; WDF, Ward Doctor Focus Group; WDI, Ward Doctor Interview; WNF, Ward Nurse Focus Group.

### 3.4 Barriers and facilitators

Using the descriptors of each Theoretical Domains Framework domain, the key issues related to implementing C4H were classified and mapped to the following nine Theoretical Domains Framework domains.

#### 3.4.1 Knowledge

There was a low level of parent knowledge about the existence of the C4H process with most parents and children being unaware of C4H prior to the interview. Once informed, they all expressed positive views. Nearly, all staff knew of C4H prior to interview, but there was uncertainty that the process had actually commenced in the hospital and about their roles.

#### 3.4.2 Skills

All parents and children identified that parents had the skills to recognize what is not normal behaviour or appearance for their child. Staff agreed that parents could recognize when their child deteriorated. Ward nurses were confident in their skills to be able to inform parents and all disagreed with the offer of developing a script (prepared wording) to assist them.

#### 3.4.3 Belief about consequences

There was agreement by all groups that the anticipated outcome from the C4H process will be to add value to patient safety monitoring and early recognition of clinical deterioration. In contrast, there was concern by all about the potential for inappropriate calls for other reasons. This may result in overuse of resources, in particular an increase in the number of MET calls and the time costs for the ICU staff. In addition, staff, in particular ward nurses, described how they would be concerned about the potential professional repercussions if they had missed signs of clinical deterioration.

#### 3.4.4 Beliefs about capabilities

There was general acceptance that parents overall had the capability and confidence to take appropriate action to escalate care. Yet doubt was also expressed, with parents and staff providing examples from their own experiences.

#### 3.4.5 Social/professional role and identity

All parents reported they would take on the role to escalate care even if that resulted in upsetting hospital staff. Nurses considered that enabling parents to use the process of C4H was aligned with their family‐centred care philosophy and practice. All participant groups identified C4H may have negative effects on parent‐staff relationships. Further some ward nurses identified that C4H may be beneficial when their own attempts to escalate care were not acted upon. Overall staff were concerned that C4H may undermine professional boundaries, causing distrust and undermine the “system” of communication between parents and staff as well as between doctors and nurses.

#### 3.4.6 Memory, attention and decision processes

To receive information about C4H, parents recommended the use of multiple communication strategies, including methods to relay information before they arrived to hospital. Staff supported the need to inform parents prior to admission and were concerned about adding to parents’ cognitive load at a time when they considered parents to already be experiencing high stress.

#### 3.4.7 Motivation and goals

In the situation when they were concerned about their child's deteriorating condition, parents described how they would always act in their child's best interest. This was identified by some parents as being a strong enough facilitator to enable them to speak up and question staff caring for their child. All groups agreed with the principle and intent of the C4H process.

#### 3.4.8 Environmental context and resources

All groups identified that a benefit of using the C4H was that the child will be reviewed by ICU staff, irrespective of whether or not that resulted in the patient being transferred to the ICU. Some parents recognized that it may be too difficult to actually speak up in the hospital environment to question ward staff. An important environmental implementation barrier for staff was the lack of clear support by the hospital executive team. This leadership group had a strong influence on staff who all agreed that C4H had not been promoted from the top down. Parents who did not speak English were recognized by staff and parents to face even greater barriers although interestingly this view was not supported by the non‐English‐speaking parent.

#### 3.4.9 Behavioural regulation

The final C4H issue identified by parents and staff was that this process presented a challenge for parents to bypass the traditional hierarchy and culture in the health‐care system. No alternatives to ways of working were identified.

## 4 DISCUSSION

Our findings describe the complexity of implementing an intervention that appears to be simple and shows how factors such as interpersonal communication, organizational hierarchy and culture or social codes of conduct can influence implementation. The key facilitator was that C4H was viewed as a positive step that could enhance patient safety. Key barriers identified were the low level of awareness by parents, lack of nursing and medical leadership support, concern about the potential increase in resource utilization, the potential negative effect on relationships, staff protecting parents from being overburdened with information and the challenge to the traditional hospital system culture. Implementation strategies can now be developed to target these barriers. There is increasing recognition about the complexity of this topic, and the need to understand which interventions are effective is now the subject of a Cochrane systematic review.^28^

Major strengths of this study were the multiple sources of data to understand and explain the implementation issues from the perspectives of children, parents and staff. The audit findings conducted on two occasions after the initial introduction of C4H revealed an extremely low level of parent awareness. Knowing this information, it was interesting to identify through analysing patient records of MET call cases that there was parent involvement in escalation of care for 11/62 (17%) MET events. More specifically, there were five occasions when parent concern had actually been the trigger for nurses to place a MET call. These events/behaviours had occurred without parents being aware of the C4H process.

To understand why parents remained unaware of the C4H process and examine issues related to implementation, four children, 13 parents and over 90 nurses and doctors were interviewed. The interviews and focus groups covered the domains of the Theoretical Domains Framework^15^ to systematically assess implementation and other behavioural factors that may have impacted on the C4H implementation. The qualitative findings explained how the low level of parent awareness may have been related to the use of passive implementation strategies and a low level of nursing and medical leadership support, key to changing organizational behaviours.^29^, ^30^ All groups of participants expressed apprehension about the potential increase in the number of MET calls and subsequent impact on health services and use of resources. This had only been reported previously as a staff concern.^11^

Other barriers to implementing a process for parent escalation of care included some that have been previously reported^9^, ^10^ and new knowledge about nurse behaviours in filtering information to avoid burdening parents. This resulted in C4H information not being prioritized when nurses provided information to parents. Difficulties in attempts to bypass the hierarchy and traditional culture in the hospital system are already well‐recognized barriers to health professionals especially nurses effectively utilizing RRS,^31^, ^32^, ^33^, ^34^ but had not been previously reported in the context of parent involvement. This is an ironic finding given that the basis and impetus for implementing a process for parent escalation of care is to reduce the delays related to organizational hierarchical behaviours and poor health professional communication.^30^, ^35^, ^36^

The study also revealed a number of facilitators. The concept of involving parents in escalation of care was viewed favourably by all participants. Interestingly, the patient record review identified that a number of parents had been actively involved in escalating care and had unknowingly followed the C4H steps. This provides a positive reinforcement that the process itself, if fully implemented, appears feasible and appropriate. Further supporting the concept of parent escalation of care, there was agreement C4H provided an added safety net for early recognition of deterioration that parents can recognize what is not normal for their child and can notice subtle changes that nurses and doctors may not detect.^14^ This parent role has yet to be evaluated in practice.^2^, ^3^

All participant groups recognized key C4H barriers and facilitators. Finding a low level of parent awareness about C4H reinforces the importance of carefully selecting and utilizing evidenced‐based implementation strategies. We examined the level of parent involvement in the C4H steps. Measuring the number of parent escalation of care calls (C4H Step 5) may not be a useful or sensitive evaluation metric.^14^ A low number of calls from parents may indicate a highly functioning health‐care system where no assistance is required to deal with patient deterioration or may reflect a low level of parent awareness of the process, or there may be other barriers to parent engagement. A high number of calls by parents may have revealed that the escalation of care system was working well or that the health‐care system was not working well and patient deterioration was being missed. In this study, the theoretical approach using the Theoretical Domains Framework to collect and analyse multiple sources of data resulted in a clear understanding of the specific issues impacting on implementation of C4H in a children's hospital setting. The most appropriate behaviour techniques can now be selected to address the key issues identified.^19^

Limitations include the low level of parent awareness about C4H meant that no parent had knowingly utilized the C4H steps, nor had nurses or doctors been involved in responding to C4H to be able to provide feedback on their experiences. There was the possibility of missing data due to the retrospective record review data collection method. It may be that there was more parent involvement in escalation of care than had been documented. The convenience sampling of parent and child participants prevented data saturation being confirmed in those groups.

## 5 CONCLUSION

This study used multiple methods to evaluate the introduction of C4H, a process to enable parent escalation of care in a children's hospital setting. Although the concept was supported in principle by children, parents and health professionals, there was a low level of awareness about C4H in practice. Initial implementation strategies had primarily targeted policy change without taking into account the challenge to practise and organizational behaviour that C4H presents. Using a theoretical approach has enabled identification of the key human and system factors of lack of high‐level management support and resourcing of the Rapid Response Systems and ineffective information sharing that influenced implementation to guide more targeted strategies.
