# Supplementary material for: Barriers and facilitators to implementing a process to enable parent escalation of care for the deteriorating child in hospital
Source: Health Expect. 2018 Jul 2;21(6):1095–103. doi: 10.1111/hex.12806 (PMC6250884; doi:10.1111/hex.12806)
Supplement: Supplementary file 1 [file HEX-21-1095-s001.docx]

**PARTNER Project Interview Guide (parents)**

***Explanation***

Thank you for agreeing to speak with me today about parents calling a MET call – the process is called “Calling for Help”. This is a new process at xx and this research is to find out how to make it work best for parents and patients. There may appear to be overlap between questions but each question is worded to obtain specific information and therefore you may find that answers are repeated. It is important to note that there are no right or wrong answers to the questions and that no one will know what your specific answers were.

The interview should take approximately 30 minutes and will be audio-recorded to ensure that all key points are accurately documented. Any identifying information (for example the names of other individuals) that you use in the course of our discussion will be removed from the interview transcripts. If you wish to end the interview before I have asked all of the questions or if you wish to withdraw from the study you are free to do so.

Do you consent to this interview being audio-recorded? Yes / no

**Background**

- - Male or Female
  - Age range- <30, 31-40, 41-50, 51-60, 61+
  - What experience have you had at the hospital? – number of admissions for your child, PICU admissions, previous MET calls/ Code Blue

For the rest of the interview I have some more specific questions about Parents calling a MET.

**Knowledge**

Are you aware of the process for parents to get help if you are worried that your child has an urgent medical concern/ his or her condition is deteriorating? Show brochure

If yes, can you tell me what you understand?

Have you had any experience in calling?

If yes can you tell me about it?

Do you feel as though you have received a sufficient information about it?

If no, what would you like to know more about?

How were you told about/ aware of it?

How would you prefer to find out about it?

**Social / Professional Role & Identity**

Do you think this hospital supports parents calling for help?

**Beliefs about Capabilities**

How easy or difficult will it be for you to call for help for your child?

What will make it easy or difficult?

Do you feel confident in hospital to know your child is getting sicker

Do you feel confident in hospital to call for help?

**Beliefs about consequences**

What are the benefits? (prompts: patients, yourself)

What are the negative aspects? (prompts: patients, yourself)

When would you consider calling for help necessary/unnecessary?

**Optimism**

In your opinion, how likely is it will lead to better care for your child?

**Environmental context and resources**

Are there any things that would help you be able to call for help?

Eg brochure

**Social influences**

Your thoughts about how would the staff on your ward respond to you raising your concerns & this influencing your decision to call for help?

If yes, how?

**Emotion**

Do you have any strong feelings about parents calling for help in this way?

Does it worry or concern you?

**Goals**

Do you want parents to be able to call for help?

Anything you’d like to tell us

Thank you

We are planning to speak to staff again in approximately 6 months when we have made changes to the Calling for Help.

Will you be happy to be interviewed again? Yes / No
